# Chronic lymphocytic leukemia presenting with ascites diagnosed by clonality analysis via gene rearrangement assay: A case report

**DOI:** 10.3892/ol.2014.2044

**Published:** 2014-04-08

**Authors:** YEN-MIN HUANG, LEE-YUNG SHIH, PO DUNN, PO-NAN WANG, MING-CHUNG KUO, JIN-HOU WU, TUNG-LIANG LIN, TZUNG-CHIH TANG, HUNG CHANG, HSIAO-WEN KAO, HSUAN-JEN SHIH, YU-SHIN HUNG

**Affiliations:** 1 Department of Internal Medicine, Division of Hematology and Oncology, Chang Gung Memorial Hospital, Keelung, Taoyun 33305, Taiwan, R.O.C.; 2 Department of Internal Medicine, Division of Hematology and Oncology, Chang Gung Memorial Hospital, Linkou, Taoyun 33305, Taiwan, R.O.C.; 3 School of Medicine, College of Medicine, Chang Gung University, Taoyuan 333, Taiwan, R.O.C.

**Keywords:** chronic lymphocytic leukemia, gene rearrangement, ascites

## Abstract

The diagnosis of chronic lymphocytic leukemia (CLL) presenting with ascites is predominantly based on the morphological and immunophenotypic characteristics, which are comparable to peripheral blood and bone marrow cells. However, it is relatively difficult to diagnose CLL due to the pleomorphism of the lymphocytes in ascites. The current study presents an 80-year-old male with a prior diagnosis of CLL who developed large ascites. Predominant T lymphocytes rendered morphological and immunophenotypic diagnosis difficult. Clonality analysis of immunoglobulin (Ig) gene rearrangements was performed on the lymphocytes from the ascites to diagnose the involvement of CLL, a laparotomy and biopsy from the peritoneal node confirmed the involvement of small lymphocytic lymphoma/CLL. The clonality analysis of Ig gene rearrangements may provide a powerful and accurate method for diagnosing CLL presenting with ascites.

## Introduction

Chronic lymphocytic leukemia (CLL) is one of the chronic lymphoproliferative disorders that is predominantly diagnosed in the elderly. Annually, >100 cases of CLL are diagnosed in Taiwan, R.O.C. (1), with ~25% of patients initially asymptomatic at diagnosis and referred to the clinic due to an abnormal white blood cell count (WBC). The common clinical presentations of CLL are B symptoms, lymphadenopathy, splenomegaly, hepatomegaly, skin lesions and membranoproliferative glomerulonephritis. CLL presenting with ascites is a rare complication, however, it has previously been reported in the literature (2–4).

The diagnosis of CLL presenting with ascites is determined when other etiologies of ascites are excluded. In a previous study, morphological and immunophenotypic characteristics were used for diagnosis (5); however, a large number of clonal cells are required in order to identify these characteristics. When the ascites contain a large number of inflammatory cells, diagnosis of CLL is difficult. In the present study, polymerase chain reaction (PCR)-based gene rearrangement was used to identify CLL presenting with ascites. The current findings indicate that this technique serves as a more powerful approach and an effective method for diagnosing ascites in patients with CLL. Patient provided written informed consent.

## Case report

The current case report presents an 80-year-old male with a history of hypertension who underwent medical treatment for 15 years. Leukocytosis was identified three years ago during an annual health examination and the patient was referred to Chang Gung Memorial Hospital (Linkou, Taiwan, R.O.C.). The WBC count was 71.2×10^9^ cells/l with lymphocyte predominance (lymphocytes, 78.7%; atypical lymphocytes, 5.7%; segments, 12.7%; monocytes, 2.2%; and eosinophils, 0.7%). The patient’s hemoglobin level and platelet count was 12.1 g/dl and 199×10^9^ cells/l, respectively. Whole body computed tomography (CT) revealed multiple small lymphadenopathy bilaterally in the neck, in the lung hilum, celiac trunk, para-aortic area, as well as splenomegaly with an enlarged splenic node. Immunophenotyping of lymphocytes in the peripheral blood revealed cluster of differentiation (CD)5(+), CD20(+) and CD23(+), which was compatible with B-cell CLL. A bone marrow biopsy revealed diffuse interstitial infiltrates of small lymphocytes, which accounted for 72.5% of the bone marrow smear. Trisomy 12 and del (17p) were detected by fluorescent *in situ* hybridization and the patient was diagnosed with CLL, Rai stage II and Binet stage B.

One year after diagnosis, 2 mg chlorambucil was administered twice daily due to progressive lymphocytosis (163.5×10^9^ cells/l with 90% lymphocytes). The WBC count and differentials had returned to the normal range following 11 months of chlorambucil treatment. However, 18 months after chlorambucil treatment, the patient developed progressive abdominal distention, which was painless, without B symptoms. Complete blood counts were as follows: Hemoglobin, 11.5 g/dl; platelet count, 106×10^9^ cells/l; WBCs, 7.8×10^9^ cells/l; segments, 63%; lymphocytes, 30.8%; monocytes, 5.3%; eosinophils, 0.6%; and basophils, 0.3%. The level of creatinine and albumin was 0.94 mg/dl and 3.57 g/dl, respectively. The electrocardiogram was normal and the cardiac sonography revealed adequate left ventricular function. Liver cirrhosis was excluded by abdominal sonography and the viral markers of hepatitis B and C were negative. The cells in ascites were predominantly lymphocytes (red blood cells, 1.285×10^9^ cells/l; WBCs, 0.710×10^9^ cells/l; neutrophils, 17%; and lymphocytes, 83%). The serum-ascites albumin gradient (SAAG) was 1.7, indicating transudative ascites. The ascites culture was negative for bacteria and tuberculosis. An abdominal CT scan showed enlarged mesenteric nodes with a progressive change of mesenteric inflammatory disease compared with the CT results at diagnosis. These findings did not exclude peritonitis.

**Figure 1:**
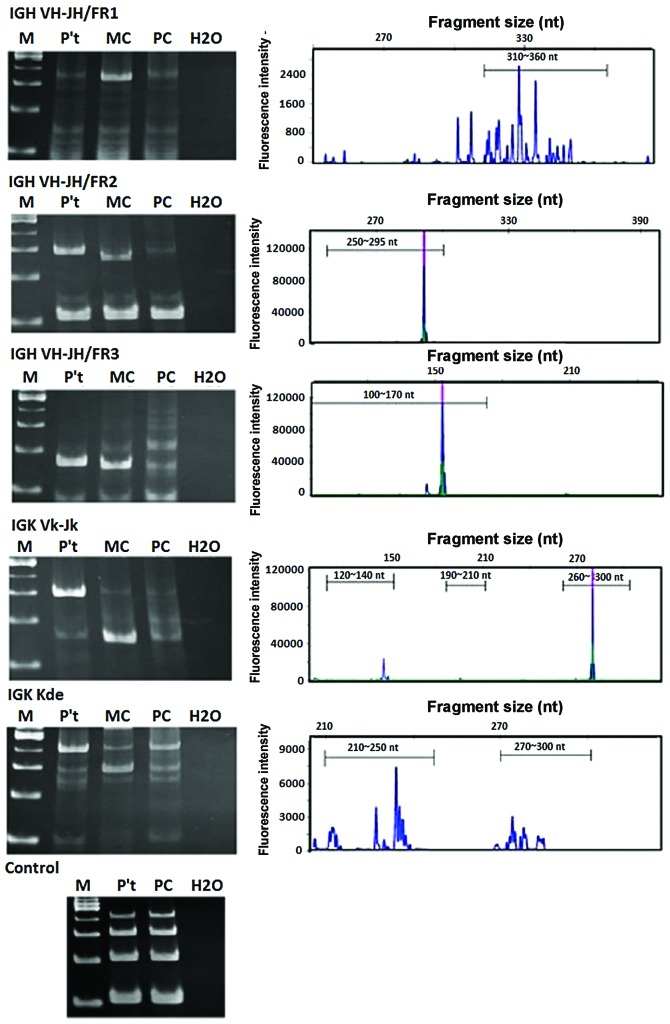
Gene rearrangement assay of ascitic cells. Presence of a clonal band for IGH VH-JH/FR2, IGH VH-JH/FR3 and the Igκ Vκ-Jκ gene by heteroduplex analysis is a poitive indication for monoclonal B cells in the ascites. There was no monoclonal band detected for IGH VH-JH/FR1 and the Igκ κde gene. M, multiple products; P’t, patient; MC, monoclonal; PC, polyclonal; H_2_O, water for negative control.

Immunophenotypic analysis of the cells in ascites showed that 80% of the cells were lymphocytes, and T and B cells accounted for <5%. The immunoglobulin (Ig) gene rearrangements analysis using the BIOMED-2 PCR protocol (6) to determine the clonality status of B cells revealed positive monoclonal B cells in the ascites. The presence of a clonal band for IGH VH-JH/FR2, IGH VH-JH/FR3 and the Igκ Vκ-Jκ genes were positive for monoclonal B cells in the ascites (Fig. 1). An explorative laparoscopy was performed to exclude peritonitis, second malignancy or large cell transformation, and large ascites with multiple white small lymph nodes over the peritoneum were identified. Biopsy of the peritoneal lymph node revealed lymphoproliferation by hematoxylin and eosin staining. Further immunohistochemical staining was positive for CD20, CD5, CD23 and negative for CD10 and cyclin D1. These findings were compatible with CLL involving the peritoneum. The patient subsequently received prednisolone and chlorambucil therapy, and the ascites rapidly regressed and disappeared after one month.

## Discussion

The first study to describe CLL presenting with ascites was in 1965 (7). Patients with CLL presenting with ascites have a short survival time (2,8). In addition, CLL presenting with chylous (9,10) and hemorrhagic (8) ascites have also been reported, as well as portal hypertension, which was identified in four cases (2,8,11,12) in which lymphocytic infiltration was considered as the etiology and resulted in transudative ascites. Additional studies have also reported exudative ascites (3,5). The difference in albumin gradient between the studies may be attributed to the various types of pathophysiology. Lymphocytic infiltration and portal hypertension may cause transudative ascites. Furthermore, peritoneal CLL involvement, which affects absorption of lymphatic ascites, increases net capillary fluid-production and may result in exudative ascites (5). However, the number of available studies are limited and it is difficult to determine the etiology of CLL using the albumin gradient.

In the present case, the patient’s SAAG was 1.7, which classified the ascites as transudates (13). The etiology of transudative ascites (SAAG ≥1.1 g/dl), including cirrhosis, alcoholic hepatitis, heart failure, massive hepatic metastases, constrictive pericarditis, Budd-Chiari syndrome and infection, were excluded by serial examinations. The cultures for bacteria and tuberculosis showed negative results, and the cytology identified mesothelial cells, macrophages, neutrophils and abundant small lymphocytes (>70%). However, the majority of the lymphocytes in ascites were T and B cells that accounted for <5%, and exhibited no evidence of light-chain restriction. In a patient with decompensated liver cirrhosis without CLL, the ascitic lymphocytes were predominantly T rather than B cells (14), and were distributed in the peripheral blood. For patients with peritoneal malignancy and ascites, tumor-infiltrating lymphocytes and T regulatory cells may contribute to the majority of T cells in ascites. The cytological findings of our patient did not support a diagnosis of CLL involvement. The traditional method used to diagnose the etiology of ascites is explorative laparotomy.

Multiplex PCR assays have been developed and standardized for the detection of clonal Ig and T cell receptor genes (15). PCR assessment of clonal Ig gene rearrangement was adopted as an important diagnostic tool in mature B-cell neoplasms (16). In the study, 56 patients exhibiting B-cell CLL were enrolled, of these, 54 (96%) cases showed a Vκ-Jκ rearrangement and 34 (61%) cases showed either a Vκ-κde or intron RSS-κde rearrangement (16). Igκ rearrangements were detectable in all of the cases studied. Therefore, this is considered to be a powerful tool for the diagnosis of B-cell CLL. The EuroClonality (BIOMED-2) consortium summarized the important pre- and post-analytical aspects of clonality testing, providing a guideline for the interpretation of clonality testing results (6). According to this protocol, the findings of our case were positive regarding clonality (generally multiple clonal results). Compared with a laparotomy, the PCR-based gene rearrangements analysis is a less invasive diagnostic approach.

In conclusion, the detection of leukemic cells in ascites may explain the formation of large ascites found in the patient in the present study. However, the interpretation of clonality of leukemic cells in ascites should be correlated with the clinical condition. To the best of our knowledge, this is the first study to detect CLL cells in ascites by clonality analysis using a gene rearrangement assay. Further clinical evidence of the application of this non-invasive technique is required.
